# Does the admission time make a difference in outcome?

**DOI:** 10.1186/cc14676

**Published:** 2015-09-28

**Authors:** André Negrão, Bruno F Mazza, Débora D da S Mazza, Sebastião César de Vasconcellos

**Affiliations:** 1 Hospital São Luiz, Rede D´Or, Unidade Morumbi, São Paulo, São Paulo, Brazil

## Introduction

The number of hospital admissions during the night is a problem in ICUs. The high occupancy rate and the high demand for beds causes the inflow of patients to occur at night in most cases. The adequacy of the health care team must be made with the intention of providing the best patient care. For this, it is important to have knowledge of hospital dynamics in the unit, so you can optimize the team for this purpose.

## Objective

The objective of this study was to evaluate whether there are differences in the outcome of patients admitted during the day compared with those admitted during the night.

## Methods

A retrospective analysis was performed from January to December 2014, using the database EPIMED^®^. We evaluated 3186 patients who were admitted to hospital ICUs. We evaluated the epidemiological characteristics of patients and the outcome of them. We classified the patients into two groups of patients: group 1 was admitted to the ICU during the day (7:00 a.m.-7:00 p.m.) and group 2 at night (7:00 p.m.-7:00 a.m.). Statistical analysis was performed using SPSS 22 software. The Student *t *test was used to analyze numerical variables and the chi-square test to analyze the categorical variables. We considered data statistically significant if *p *<0.05.

## Results

There was a higher number of admissions to the ICU at night, 2154 (67.6%) vs. 1032 (32.4%). The principal source of patients was the emergency room, 2372 (74.5%) patients. There are more clinical than surgical patients, 82.2% vs. 17.8%. There were no significant differences between groups regarding demographic variables and severity scores. The SAPS 3 score was 41.67 ± 11.44 in group 1 and 41.81 ± 12.51 in group 2 (*p *= 0.75). There was no difference in length of stay in the ICU, group 1: 4.61 ± 7.54 vs. group 2: 4.83 ± 7.23 (*p *= 0.42). There was no difference in ICU, group 1: 91 (4.2%) vs. group 2: 55 (5.6%), or hospital, group 1: 164 (8.2%) vs. group 2: 98 (10.4%), mortality between groups, *p *= NS.

## Conclusion

In our study, we found that a high admission rate occurs at night. The ICU team needs to be adjusted for this demand. There is no difference between groups in severity, length of stay and ICU or hospital mortality in this group of patients.

